# Breast cancer in Zimbabwe: patterns of care and correlates of adherence in a national referral hospital radiotherapy center cohort from 2014 to 2018

**DOI:** 10.1002/cam4.3764

**Published:** 2021-05-10

**Authors:** Shekinah Nefreteri Cluff Elmore, Melinda Mushonga, Hari Subramaniam Iyer, Caroline Kanda, Shirley Chibonda, Fallon Chipidza, Rudo Makunike Mutasa, David Muchuweti, Edwin G. Muguti, Aspect Maunganidze, Ntokozo Ndlovu, Jennifer Ruth Bellon, Anna Mary Nyakabau

**Affiliations:** ^1^ Harvard Radiation Oncology Program Boston USA; ^2^ Harvard Medical School Boston USA; ^3^ Department of Radiation Oncology University of North Carolina Chapel Hill USA; ^4^ Parirenyatwa Hospital Radiotherapy and Oncology Center Harare Zimbabwe; ^5^ Dana‐Farber Cancer Institute Boston USA; ^6^ Department of Radiation Oncology Dana‐Faber Cancer Institute/Brigham and Women’s Hospital Boston Massachusetts USA; ^7^ Department of Pathology University of Zimbabwe College of Health Sciences Harare Zimbabwe; ^8^ Department of Surgery University of Zimbabwe College of Health Sciences Harare Zimbabwe; ^9^ Department of Oncology University of Zimbabwe College of Health Sciences Harare Zimbabwe; ^10^ Cancerserve Trust Harare Zimbabwe

**Keywords:** adherence to care, Breast cancer, global health, multidisciplinary team, radiation oncology, radiotherapy, resource‐limited setting

## Abstract

**Background:**

Breast cancer is the second most common cancer among women in Zimbabwe. Patients face socioeconomic barriers to accessing oncology care, including radiotherapy. We sought to understand patterns of care and adherence for women with breast cancer in sub‐Saharan Africa (SSA) with radiotherapy access.

**Methods:**

A retrospective cohort was created for women with breast cancer evaluated at the Parirenyatwa Hospital Radiotherapy and Oncology Center (RTC) from 2014 to 2018. Clinical data were collected to define patterns of care. Non‐adherence was modeled as a binary outcome with different criteria for patients with localized versus metastatic disease.

**Results:**

In total, 351 women presented with breast cancer with median age 51 at diagnosis (IQR: 43–61). Receptor status was missing for 71% (248). 199 (57%) had non‐metastatic disease, and 152 (43%) had metastases. Of women with localized disease, 34% received post‐mastectomy radiation. Of women with metastatic disease, 9.7% received radiotherapy. Metastatic disease and missing HIV status were associated with increased odds of study‐defined non‐adherence (aOR: 1.85, 95% CI: 1.05, 3.28; aOR: 2.13, 95% CI: 1.11, 4.05), while availability of ER/PR status was associated with lower odds of non‐adherence (aOR: 0.18, 95% CI: 0.09, 0.36).

**Conclusions:**

Radiotherapy is likely underutilized for women with breast cancer, even in a setting with public sector availability. Exploring patient‐level factors that influence adherence to care may provide clinicians with better tools to support adherence and improve survival. Greater investment is needed in multidisciplinary, multimodality care for breast cancer in SSA.

## Graphical abstract

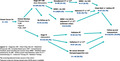

## 1 INTRODUCTION

Breast cancer is the most common cancer among women worldwide.^1^, ^2^ While incidence is lower in low‐ and lower‐middle income countries (LLMIC) than in upper‐middle‐ and high‐income countries (UMC/HIC), age‐standardized mortality is higher.^1^, ^3^, ^4^, ^5^ Women in LLMIC are more likely to present with locally advanced or metastatic disease and are less likely to have access to diagnostic and therapeutic options concordant with international guidelines.^4^, ^5^, ^6^, ^7^, ^8^, ^9^, ^10^, ^11^, ^12^, ^13^, ^14^ While interest in addressing breast cancer inequities in LLMIC is growing, research surrounding their causes remains limited, particularly in sub‐Saharan Africa (SSA).^4^, ^15^ Few clinical cohorts with detailed clinical and treatment data have been reported, particularly in settings with access to radiotherapy.^8^, ^11^, ^12^, ^13^, ^14^, ^15^, ^16^

In Zimbabwe, breast cancer is the second most common cancer among women. There were an estimated 2000 incident cases and more than 900 deaths in 2018, though only a fraction of these patients are documented as presenting to care with a breast cancer diagnosis.^1^, ^2^ The full complement of specialized oncology services is available between the two largest cities, Harare and Bulawayo.^16^, ^17^ However, patients face social and economic difficulties in access to care, including significant delays, and public systems are often stretched both financially and logistically.^17^ The goal of the current research was to understand the presenting characteristics, adherence to care, and gaps in service delivery for women with breast cancer in a sub‐Saharan African setting with access to radiotherapy. To explore this, the authors developed a retrospective cohort of all women with a diagnosis of breast cancer who were evaluated at the Parirenyatwa Hospital Radiotherapy and Oncology Centre in Harare, Zimbabwe from 2014 to 2018.

## 2 METHODS

A retrospective cohort was created including all women with a pathologic diagnosis of breast cancer who were at least 18 years of age and were evaluated at the Parirenyatwa Hospital Radiotherapy and Oncology Center (RTC) in Harare, Zimbabwe from 2014 to 2018. The RTC is the major public oncology referral center in the country, providing both systemic therapy and radiotherapy services. Patients from the private sector who were referred to RTC for radiotherapy services only were excluded as their records were incomplete. Paper medical charts were reviewed and information on demographics, presenting symptoms, pathology, clinical staging, treatment, and follow‐up was abstracted. Verification of radiotherapy plans was confirmed in the electronic treatment and verification system (Eclipse, Varian Medical Systems, Palo Alto, USA) as needed. Abstracted data was entered electronically using the XLS form standard with Open Data Kit (Open Data Kit, Ona Systems, Nairobi, Kenya) on Android tablets. Forms included built‐in data validity checks. Further data checks (e.g., date validity) were instituted following abstraction, and records with potential errors were re‐reviewed. Travel time from each provincial referral hospital to Parirenyatwa hospital, a proxy for geographic accessibility to breast cancer care, was estimated using geographic information systems and the Access Mod 5 algorithm.^18^ We modeled travel time using categories (0 minute (referred from Harare province), 0–120 minute, 121–360 minute, >360 minute). Full details about the methodology are provided in the Supplementary Materials.

Statistical analyses were performed with SAS 9.4 (SAS, Cary, North Carolina USA). We summarized cohort characteristics using medians and interquartile range for continuous variables and proportions for categorical and binary variables. Maps were made using ArcMap 10.6 (Esri, Redlands, California, USA) with population denominators estimated using the number of women in each region based on the most recent census.^19^

We sought to identify patient factors associated with adherence to guideline‐concordant treatment per the National Comprehensive Cancer Network (NCCN) guidelines.^20^ As full data on guideline‐concordant care was not available given the retrospective nature of the study, we defined non‐adherence as (a) the receipt of breast surgery without any neoadjuvant or adjuvant therapy, for patients with locally advanced disease or (b) the receipt of no therapy for patients with metastatic disease after physician recommendation and modeled as a binary outcome. We selected the following covariates: age, HIV status (positive/negative/unknown), availability of ER/PR status, health insurance (yes/no), diagnosis year (binary: prior to 2017 inclusive/post‐2017, corresponding to the beginning of macroeconomic instability), referral travel time to Parirenyatwa hospital, and presence of metastasis at diagnosis (yes/no).^21^, ^22^ Covariates were chosen based on completeness of data and prior literature.^8^, ^15^, ^17^, ^23^, ^24^, ^25^, ^26^, ^27^ We estimated odds ratios and 95% confidence intervals using multiple logistic regression and reported the c‐statistic and Hosmer‐Lemeshow test for goodness‐of‐fit.

All necessary ethical approvals were obtained, including approval from the Medical Research Council of Zimbabwe (Harare, Zimbabwe) and Partners Healthcare (Boston, Massachusetts, USA).

## 3 RESULTS

### 3.1 Patient demographics

**FIGURE 1:**
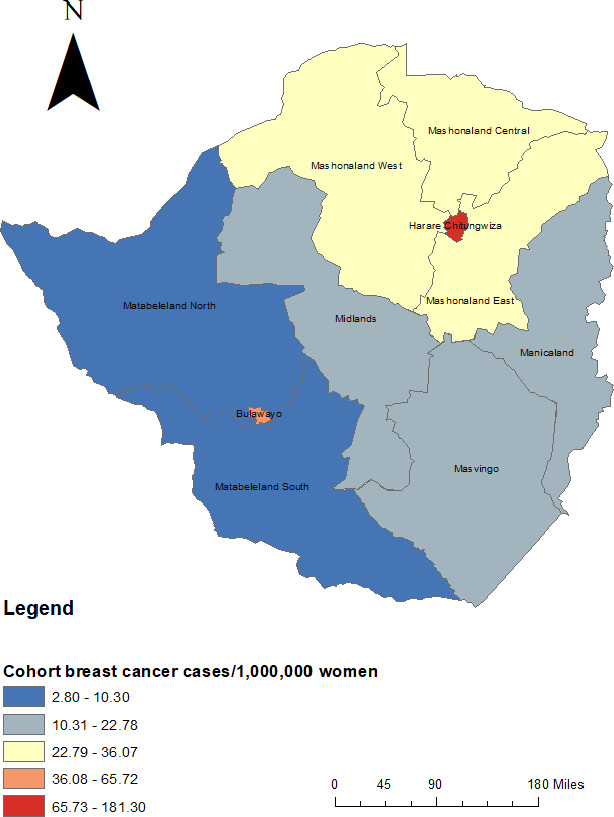
Map of Zimbabwe with cohort geography.

There were 351 women included in the cohort. All were identified as of black/African race/ethnicity. Full demographics, breast cancer risk factors, and breast cancer characteristics are reported in Table 1. The median age at time of diagnosis was 51 years (IQR: 43–61). Median body mass index (BMI) at intake was 28.6 (IQR: 24.5–33.2), and Karnofsky Performance Status (KPS) was 80–100% for most patients (43.3%). A minority (51, 14.5%) were HIV infected, 207 (59%) were HIV uninfected, and 93 (26.5) had unknown HIV status. A minority (71, 20.2%) had health insurance coverage. Most (199, 56.7%) provide a contact address located in Harare Province, though this may have been a relative's address in some cases. Cohort geography is detailed in Figure 1. The map suggests that most cohort participants came from Harare province and surrounding provinces, with fewer participants from the western provinces.

**TABLE 1:** Descriptive characteristics of harare breast cancer cohort.

	N (%) or median [IQR]
Age at diagnosis∞	51.3 [43.4–61.4]
BMI at intake∞*	28.6 [24.5–33.2]
KPS at intake	
80–100%	152 (43.3)
60–70%	30 (8.6)
40–50%	8 (2.3)
HIV status	
Infected	51 (14.5)
Uninfected	207 (59)
Unknown	93 (26.5)
Age at menarche∞**	14 [13–15]
No. children∞***	3 [2–5]
History of hormonal birth control use	
Yes	201 (57.3)
No	53 (15.1)
Unknown	97 (27.6)
History of breast feeding	
Yes	210 (59.8)
No	16 (4.6)
Unknown	125 (35.6)
Family history of cancer	
Yes	101 (28.8)
No	179 (51)
Unknown	71 (20.2)
Health Insurance Coverage	71 (20.2)
Residence	
Harare province	199 (56.7)
Bulawayo province	23 (6.6)
Other	129 (36.8)
Referral time	
0 min (Harare province)	199 (56.7)
0–120 min	46 (13.1)
121–360 min	65 (18.5)
>360 min	41 (11.7)
First Presenting Signs/Symptom(s)****	
Breast lump	272 (67.5)
Breast or arm swelling	23 (5.7)
Breast discomfort	14 (3.5)
Underarm Fullness	9 (2.2)
Nipple discharge	7 (1.7)
Nipple inversion	5 (1.2)
Skin changes or redness	7 (1.7)
Headache	4 (1)
Weakness	2 (0.5)
Other	24 (6)
Not documented	36 (9)
AJCC 2018 anatomic stage at diagnosis	
IIA	5 (1.4)
IIB	6 (1.7)
IIIA	12 (3.4)
IIIB	33 (9.4)
IIIC	9 (2.6)
Non‐metastatic, sub‐stage unknown	134 (38.2)
IV	152 (43.3)
Histology	
Invasive ductal carcinoma	253 (72.1)
Other	41 (11.7)
Unknown	57 (16.2)
Grade	
G1‐2	111 (31.6)
G3	80 (22.8)
Unknown	160 (35.6)
Receptor status	
HR+/HER2‐	47 (13.4)
HR‐/HER2‐	18 (5.1)
HR+/HER2+	15 (4.3)
HR‐/HER2+	13 (3.7)
HR+/HER2 Unknown	10 (2.9)
Unknown	248 (70.7)

Notes

All female gender, all black African race/ethnicity.

∞ (median[IQR]);

X=Unknown

* N = 178,

** N = 185,

*** N = 307,

**** N = 338;

### 3.2 Breast cancer risk factors

The median age at menarche was 14, and the median number of children was 3. Most women used hormonal birth control (201, 57.3%) and reported breastfeeding (210, 59.8%). A substantial minority reported a family history of cancer (101, 28.8%), though this was not limited to first‐degree relatives only as this was not specified in the medical records.

### 3.3 Breast cancer characteristics

Most women presented with a breast mass (272, 67.5%). More than half of women (199, 56.7%) presented with non‐metastatic disease. For most of these (134/199, 67.3%), the detailed substage could not be determined. Invasive ductal carcinoma was the most common histology (253, 72.1%). Histologic grade was unknown for 160 (35.6%), G1‐2 for 111 (31.6%), and G3 (22.8%) of patients. Receptor status was unknown for most (248, 70.7%) patients.

### 3.4 Patterns of care

**FIGURE 2:**
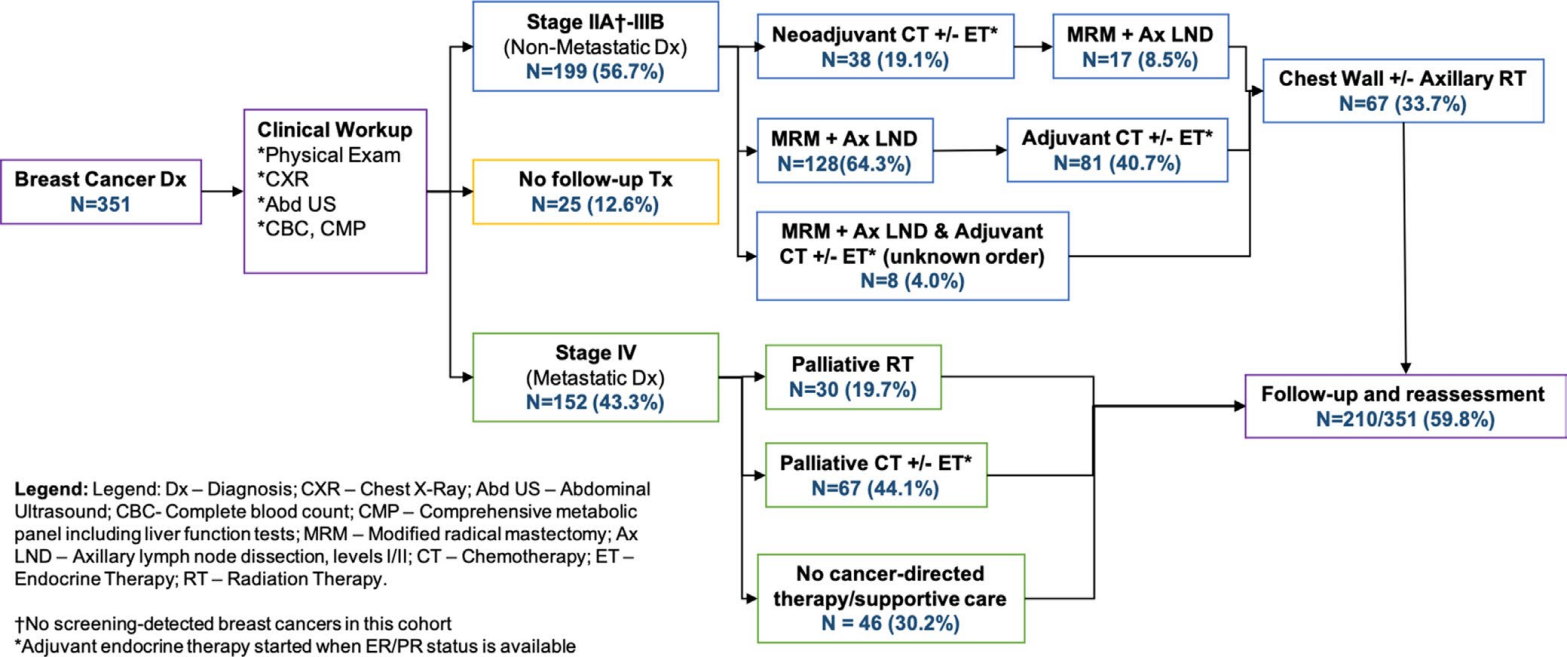
Patterns of Care 2014–2018.

Patterns of care are detailed in Figure 2. Among the 199 patients with non‐metastatic breast cancer, 25 (12.6%) were lost to follow‐up after their initial visit. 38 (19.1%) received neoadjuvant chemotherapy (CT) with or without endocrine therapy (ET) and then 17 (8.5%) received subsequent modified radical mastectomy (MRM) with axillary lymph node dissection (Ax LND). 128 (64.3%) received initial MRM with Ax LND which was followed by adjuvant CT with or without ET in 81 (40.7%). Only 67 (33.7%) of both neoadjuvant and adjuvant groups received post‐mastectomy radiation therapy (RT). ET was most commonly tamoxifen. Neoadjuvant and adjuvant CT were most commonly AC‐T (Adriamycin/doxorubicin, cyclophosphamide, and Taxol/docetaxel). Post‐mastectomy RT was most commonly delivered as 50 Gy in 25 fractions to the chest wall following CT simulation with a 2D/3D plan with 6 MV photons. Dynamic wedges and field‐in‐field techniques were commonly used to optimize plans.

Among the 152 patients with metastatic disease, 46 (30.2%) did not receive any cancer‐directed therapy and details on their receipt of best supportive care are unknown. Thirty (19.7%) received RT for any indication and 67 (44.1%) received palliative CT with or without ET. Palliative chemotherapy regimens varied but included both combination regimens and single agent taxanes.

Across the entire 351‐woman cohort, 210 (59.8%) had at least one follow‐up visit after the completion of treatment. Median follow‐up from biopsy to first treatment was 1.38 months (IQR: 0.69–3.68). Median time from biopsy to last follow‐up was 10.97 months (IQR: 2.73–18.25). Median follow‐up time from treatment completion to last‐follow‐up was 9.53 months (IQR: 2.73–16.34).

**FIGURE 3:**
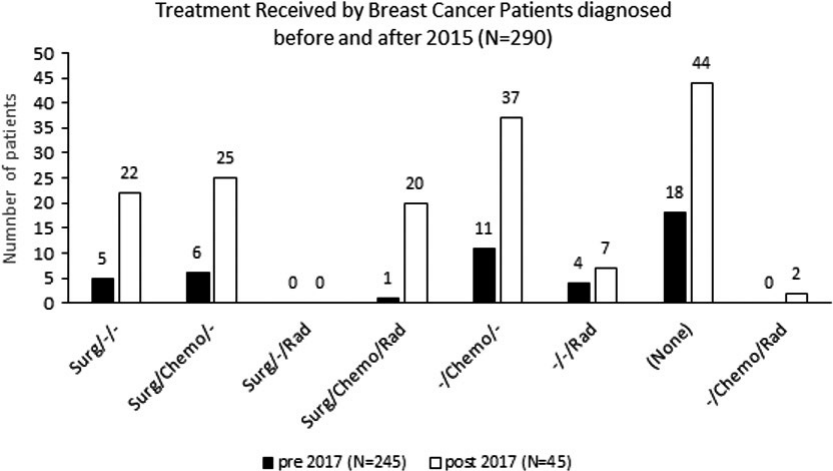
Treatment received by breast cancer study participants before and after 2017 (N = 290).

Treatment regimens received by breast cancer cohort participants over time are presented in Figure 3. There was a statistically significant difference in treatment regimens received before and after 2017 (*P* = 0.0007). Among those breast cancer study participants for whom diagnosis date was available (N = 290), participants were more likely to receive a full course of treatment (surgery, chemotherapy and radiation) before 2017 (21.6%) compared to post‐2017 (2.2%). Breast cancer patients were less likely to receive no treatment before 2017 (21.6%) compared to post‐2017 (40.0%) (*P* = 0.0084).

### 3.5 Adherence to care

Results from multiple logistic regression among the 290 participants (83%) with complete data are presented in Table 2. The c‐statistic was 0.75, and the Hosmer‐Lemeshow Goodness‐of‐fit test suggested good model fit (*P* = 0.50). Metastatic disease was statistically significantly associated with 85% increased odds of study‐defined treatment non‐adherence (aOR: 1.85, 95% CI: 1.05, 3.28). Missing HIV status was also associated with significantly higher odds of study‐defined treatment non‐adherence (aOR: 2.13, 95% CI: 1.11, 4.05). Availability of ER/PR status was associated with significantly lower odds of treatment non‐adherence (aOR: 0.18, 95% CI: 0.09, 0.36). Compared to women referred to Parirenyatwa Hospital within Harare province, those assumed to have been referred from provincial hospitals within 0–120 minute had 81% higher odds of treatment non‐adherence (aOR: 1.81, 95% CI: 0.79, 4.13), though this was not statistically significant.

**TABLE 2:** Odds ratios and 95% confidence intervals for association between patient factors and Non‐adherence to Care.

Factor	aOR (95% CI)	p‐value
Age (1‐year)	1.00 (0.98, 1.02)	0.85
HIV status		0.054
Infected	0.92 (0.40, 2.12)	0.27
Uninfected	Ref	
Unknown	2.13 (1.11, 4.05)	0.021
Referral time for provincial hospital		0.29
Same province (Harare)	Ref	
0–120 min	1.81 (0.79, 4.13)	0.058
121–360 min	0.87 (0.40, 1.91)	0.74
>360 min	0.56 (0.19, 1.68)	0.19
Diagnosis prior to 2017	0.88 (0.41, 1.89)	0.75
Insurance	0.91 (0.42, 1.97)	0.81
Availability of ER/PR status	0.18 (0.09, 0.36)	<.0001
Metastatic disease at diagnosis	1.85 (1.05, 3.28)	0.034

C‐statistic: 0.75, Hosmer‐Lemeshow Goodness‐of‐fit p‐value: 0.50

## 4 DISCUSSION

We sought to present a comprehensive and contemporary series of women with breast cancer from diagnosis to treatment from the largest public cancer treatment program in Harare, Zimbabwe. This study extends existing knowledge about breast cancer in low‐ and lower‐middle‐income countries by specifically characterizing disease characteristics, patterns of care and treatment adherence in a center with access to radiotherapy. Parallel to previously published series in the region, most women presented with locally advanced or metastatic disease.^3^, ^28^, ^29^, ^30^, ^31^, ^32^ Also like most countries in the region, Zimbabwe does not currently have a national breast cancer screening program. Thus, most breast cancer presentations will be symptomatic and inherently more likely to be associated with advanced disease. Of note, a breast cancer screening program was started and received significant demand for screening from the public. In fact, the clinic received such robust demand for screening that it outstripped the capacity to screen asymptomatic patients. A breast disease management clinic to expedite the care of symptomatic patients was created in its place and that is still operational.

Notably, only 34% of women with localized disease who underwent mastectomy with neoadjuvant or adjuvant chemotherapy received post‐mastectomy radiation. And among those presenting with metastases, a minority (19.7%) received radiotherapy for any indication. While these numbers potentially underestimate the number of patients who might have benefited from radiotherapy, they help to ascertain the radiotherapy utilization rate (RUR) for women with breast cancer in the region in the context of limited prior data. A systematic review from predominantly high‐income country (HIC) data estimated overall RUR for breast cancer to be 56–72%.^33^ RUR for patients with metastatic breast cancer near the end of life was >50% in another, multicenter HIC series.^34^ Within SSA, a retrospective breast cancer cohort from Nigeria reported that less than half of patients with non‐metastatic disease received both adjuvant chemotherapy and adjuvant radiotherapy after mastectomy.^35^ However, receipt of radiotherapy was significantly associated with improved overall survival, independent of sub‐stage. Additionally, in a preliminary analysis from Botswana, only 64% of patients with at least one indication for post‐mastectomy radiotherapy received the recommended therapy.^7^

In this cohort, among the 103 patients whose receptor status was known, majority were ER+or PR+accounting for about 68% of the cases, while 18(17%) had triple‐negative breast cancer (TNBC). Assuming these numbers to be representative of the broader cohort, this molecular breakdown is similar to other studies with patients of East and Southern African ancestry.^32^, ^36^, ^37^, ^38^, ^39^ However, hormone and HER‐2 receptor status was unknown in 71% of patients. The high out‐of‐pocket cost of immunohistochemical staining likely precluded most patients from having their tumors undergo additional testing. Zimbabwe does not currently have a national health insurance coverage program. Thus, few patients have insurance that is either privately purchased or employer sponsored. Even for patients with insurance coverage, most still have out of pocket costs. Missing receptor status adversely impacts patient outcomes by limiting risk stratification. Furthermore, with unknown distribution of molecular subtypes of breast cancer, the health system is limited in forecasting and procuring necessary systemic therapy.

The exploratory analysis of adherence to guidelines provides multiple areas for further study. Presence of metastatic disease and missing HIV infection were associated with treatment non‐adherence (aOR: 1.85 and aOR: 2.13, respectively) while known hormone receptor status was associated with increased adherence (aOR: 0.18). Contributing factors to non‐adherence to treatment for patients with metastatic breast cancer include multiple, costly steps that are often necessary prior to initiating therapy. These costs include clinical tests and the procurement of chemotherapeutic agents and other essential medications. While these delays may also be present for patients with localized disease, the delays may lead to reductions in adherence due to interim changes in functional status for patients with metastases. Further, non‐documented HIV status may be a proxy for either not being tied into existing systems of care or lack of funds to procure additional test results, both of which may lead to reduced adherence. Finally, the high cost of immunohistochemistry likely makes it unaffordable to most patients.^40^ Thus, those patients who were able to afford these immunohistochemistry results may be more able to afford and thus adhere to further care. The possible economic underpinnings of adherence are also suggested by the difference in patients who received no therapy pre‐ and post‐2017 economic crisis. This reinforces the simple conclusion that complete pathologic diagnosis leads to improved oncology care.^26^

Furthermore, patients with an estimated referral travel time in the 0–120 minute range had an 81% higher odds of treatment non‐adherence when compared to patients with an estimated 0 minute referral travel time (aOR: 1.81, 95% CI: 0.79, 4.13). Although this result was not statistically significant, odds did not appear to differ for patients who were in the 121–360 minute (aOR: 0.87, 95% CI: 0.40, 1.91) >360 minute (aOR: 0.56, 95% CI: 0.19, 1.68) referral travel time categories. Our study did not have data available about actual patient distances or daily travel times to treatment. However, one explanation of this data could be that some patients from greater distances secured housing in Harare for treatment. Or, additionally, that patients who lived very long distances from treatment may be self‐selected for higher socioeconomic status, which is also a likely correlate of adherence. Additional attention is needed to geographic considerations in future breast cancer adherence studies in sub‐Saharan Africa.

This work reinforces and expands upon conclusions from a series by Muchuweti et al 2017 looking prospectively at reasons for delay in patients with breast cancer attending the outpatient surgical clinics at Parirenyatwa Hospital from 2010 to 2013.^29^ Most women in their cohort (53/73, 73%) presented with advanced disease. Significant predictors of presentation delay included low socioeconomic status, low formal education, and lack of knowledge about breast cancer. In other series from the region, delays at the health‐system level were frequent, including delays to biopsy, initiation of surgical care, and initiation of systemic therapy.^41^, ^42^, ^43^, ^44^

Multilevel interventions are needed to improve the rate of late presentation and to enhance the multidisciplinary care that women receive once they reach oncology care.^45^, ^46^ Regarding the latter, multidisciplinary team (MDT) meetings that include all specialties involved in breast cancer care are held routinely at the Parirenyatwa Hospital. Radiology, pathology, surgery, and clinical oncology all participate in the review of patient cases and the development of recommendations. Yet, given the demands of clinical care and limited administrative resources, all patients are not reviewed through this mechanism. Expansion of the MDT paradigm to all patients who present with breast cancer may yield improved results in adherence, guideline‐concordant care, and delays as has been shown in other settings.^47^, ^48^, ^49^, ^50^ Additionally, patient navigation and financial support strategies could be implemented alongside the MDT to address systems‐level barriers to care. These initiatives will need continued funding commitments and may pay the largest dividends in improving cancer outcomes.^51^

This series is limited in that it does not include patients with localized disease who underwent mastectomy and then did not follow‐up for at least an initial visit at RTC. This loss‐to‐follow‐up in the cohort prior to treatment may bias clinical findings if patients who failed to remain in care were more likely to receive a specific type of treatment. There may also be a small sub‐group of patients here who receive a palliative mastectomy to care for ulcerating or fungating masses whose outcomes would ideally be treated separately. Yet, these patients cannot be distinguished due to the retrospective nature of the cohort and the surgical documentation not having been collected in real time. We are also unable to report long‐term follow‐up data and thus overall survival as many patients do not return to care after completing treatment. Enhanced patient tracing after the complement of treatment is an urgent need both to enhance outcomes for individual patients and to conduct meaningful research. Additionally, our definition of adherence is necessarily imprecise and does not differentiate between “partial” and “complete” adherence to recommended treatment. Future, prospective studies are needed to define and monitor adherence in real‐time to develop a better understanding of barriers and facilitators and enable intervention studies.

## 5 CONCLUSIONS

Breast cancer continues to comprise a significant burden of disease for women in sub‐Saharan Africa. This series presents a detailed analysis of patterns of care for women with breast cancer in Harare, Zimbabwe. Radiotherapy is a critical component of care for women with localized and metastatic disease. RTU and impact on outcomes like survival need further characterization. Exploring patient‐level factors that influence adherence to care may provide clinicians with better tools to support adherence and quality of life, both of which could reasonably lead to better survival. Greater investment is needed to support improved systems for breast cancer care delivery, particularly to improve adherence and reduce loss to follow‐up. Breast cancer is highly treatable and often curable. We hope that these data add to the growing literature demonstrating the urgent need to improve breast cancer diagnosis and treatment for women in sub‐Saharan Africa.

## 6 ETHICS STATEMENT

All necessary ethical approvals were obtained, including approval from the Medical Research Council of Zimbabwe (Harare, Zimbabwe) and Partners Healthcare (Boston, Massachusetts, USA).

## 7 PRECIS FOR USE IN THE TABLE OF CONTENTS

Radiotherapy is likely underutilized for women with breast cancer in Zimbabwe, despite public sector availability. Greater investment is needed in multidisciplinary, multimodality care for breast cancer in sub‐Saharan Africa.

## CONFLICT OF INTEREST

The authors report no conflicts of interest related to this work.

## AUTHOR CONTRIBUTIONS

S.N.C.E., M.M., H.S.I., C.K., S. C., F.C., R.M.K., D.M., E.G.M., A.M., N.N., J.R.B., and A.M.N. contributed to the design and implementation of the research, the interpretation of the results, and to the writing of the manuscript. H.S.I. and S.N.C.E. contributed to the primary analysis of the results.

## Supporting information

Figure S1

## References

[1] The International Agency for Research on Cancer . The Global Cancer Observatory. Published 2018. Accessed September 14, 2020. https://gco.iarc.fr/today/.

[2] Bray F , Ferlay J , Soerjomataram I , Siegel RL , Torre LA , Jemal A . Global cancer statistics 2018: GLOBOCAN estimates of incidence and mortality worldwide for 36 cancers in 185 countries. CA Cancer J Clin. 2018;68:394‐424. 30207593 10.3322/caac.21492

[3] McCormack V , McKenzie F , Foerster M , et al. Breast cancer survival and survival gap apportionment in sub‐Saharan Africa (ABC‐DO): a prospective cohort study. Lancet Glob Health. 2020;8:e1203‐e1212. 32827482 10.1016/S2214-109X(20)30261-8 PMC7450275

[4] Anderson BO , Cazap E , El Saghir NS , et al. Optimisation of breast cancer management in low‐resource and middle‐resource countries: executive summary of the Breast Health Global Initiative consensus, 2010. Lancet Oncol. 2011;12:387‐398. 21463833 10.1016/S1470-2045(11)70031-6

[5] Vanderpuye VDNK , Olopade OI , Huo D . Pilot Survey of Breast Cancer Management in Sub‐Saharan Africa. J Glob Oncol. 2017;3:194‐200. 28717760 10.1200/JGO.2016.004945 PMC5493219

[6] Vanderpuye V , Grover S , Hammad N , et al. An update on the management of breast cancer in Africa. Infect Agent Cancer. 2017;12:13. 28228841 10.1186/s13027-017-0124-y PMC5307840

[7] Buscariollo DL , Bagley A , Suneja G , et al. Postoperative radiation therapy utilization for localized breast cancer in Botswana. Int J Radiat Oncol Biol Phys. 2017;99:E391.

[8] Foerster M , Anderson BO , McKenzie F , et al. Inequities in breast cancer treatment in sub‐Saharan Africa: findings from a prospective multi‐country observational study. Breast Cancer Res. 2019;21:93. 31409419 10.1186/s13058-019-1174-4 PMC6691541

[9] O’Neil DS , Keating NL , Dusengimana JMV , et al. Quality of breast cancer treatment at a rural cancer center in Rwanda. J Glob Oncol. 2018;4:1‐11. 10.1200/JGO.2016.008672 PMC6180813 30241207

[10] Jedy‐Agba E , McCormack V , Adebamowo C , Dos‐Santos‐Silva I . Stage at diagnosis of breast cancer in sub‐Saharan Africa: a systematic review and meta‐analysis. Lancet Glob Health. 2016;4:e923‐e935. 27855871 10.1016/S2214-109X(16)30259-5 PMC5708541

[11] Jedy‐Agba E , McCormack V , Olaomi O , et al. Determinants of stage at diagnosis of breast cancer in Nigerian women: sociodemographic, breast cancer awareness, health care access and clinical factors. Cancer Causes Control. 2017;28:685‐697. 10.1007/s10552-017-0894-y. 28447308 PMC5492222

[12] Kantelhardt EJ , Zerche P , Mathewos A , et al. Breast cancer survival in Ethiopia: a cohort study of 1,070 women. Int J Cancer. 2014;135:702‐709. 24375396 10.1002/ijc.28691

[13] Obrist M , Osei‐Bonsu E , Awuah B , et al. Factors related to incomplete treatment of breast cancer in Kumasi, Ghana. Breast. 2014;23:821‐828. 25282667 10.1016/j.breast.2014.08.014 PMC4274250

[14] Coetzee WC , Apffelstaedt JP , Zeeman T , Plessis M . Disparities in breast cancer: private patients have better outcomes than public patients. World J Surg. 2018;42:727‐735. 28819769 10.1007/s00268-017-4187-0

[15] Pace LE , Shulman LN . Breast cancer in Sub‐Saharan Africa: challenges and opportunities to reduce mortality. Oncologist. 2016;21:739‐744. 27091419 10.1634/theoncologist.2015-0429 PMC4912363

[16] Ndarukwa S , Nyakabau AM , Chagpar AB , et al. American Society of Clinical Oncology multidisciplinary cancer management course: connecting lives, cancer care, education, and compassion in zimbabwe‐a pilot for efforts of sustainable benefit? J Glob Oncol. 2017;3:409‐417. 28831449 10.1200/JGO.2016.003673 PMC5560449

[17] Kadzatsa W , Ndarukwa‐Jambwa S . Breast cancer treatment in resource constrained countries: a Zimbabwean perspective. Curr Breast Cancer Rep. 2019;11:170‐174.

[18] Ray N , Ebener S . AccessMod 3.0: computing geographic coverage and accessibility to health care services using anisotropic movement of patients. Int J Health Geogr. 2008;7(1):63. 19087277 10.1186/1476-072X-7-63 PMC2651127

[19] Zimbabwe National Statistics Agency . Women and Men in Zimbabwe Report 2012. Zimbabwe National Statistics Agency. 2013.

[20] National Comprehensive Cancer Network . NCCN Clinical Practice Guidelines in Oncology (NCCN Guidelines). Breast Cancer. 2020.

[21] The World Bank . Zimbabwe Economic Update: The State in the Economy. Published July 1, 2017. Accessed September 15, 2020. https://www.worldbank.org/en/country/zimbabwe/publication/zimbabwe‐economic‐update‐the‐state‐in‐the‐economy

[22] The World Bank . The World Bank In Zimbabwe: Overview. Published October 13, 2019. Accessed September 15, 2020. https://www.worldbank.org/en/country/zimbabwe/overview

[23] Joko‐Fru WY , Miranda‐Filho A , Soerjomataram I , et al. Breast cancer survival in sub‐Saharan Africa by age, stage at diagnosis and human development index: a population‐based registry study. Int J Cancer. 2020;146:1208‐1218. 31087650 10.1002/ijc.32406 PMC7079125

[24] Cubasch H , Ruff P , Joffe M , et al. South African breast cancer and HIV outcomes study: methods and baseline assessment. J Glob Oncol. 2017;3:114‐124. 28706996 10.1200/JGO.2015.002675 PMC5493271

[25] Anyanwu SNC , Egwuonwu OA , Ihekwoaba EC . Acceptance and adherence to treatment among breast cancer patients in Eastern Nigeria. Breast. 2011;20(Suppl 2):S51‐S53. 21295480 10.1016/j.breast.2011.01.009

[26] Martei YM , Pace LE , Brock JE , Shulman LN . Breast cancer in low‐ and middle‐income countries: why we need pathology capability to solve this challenge. Clin Lab Med. 2018;38:161‐173. 29412880 10.1016/j.cll.2017.10.013 PMC6277976

[27] Spano J‐P , Lanoy E , Mounier N , Katlama C , Costagliola D , Heard I . Breast cancer among HIV infected individuals from the ONCOVIH study, in France: therapeutic implications. Eur J Cancer. 2012;48:3335‐3341. 22766516 10.1016/j.ejca.2012.05.019

[28] Fadelu T , Damuse R , Lormil J , et al. Patient characteristics and outcomes of nonmetastatic breast cancer in haiti: results from a retrospective cohort. Oncologist. 2020;25:e1372–e1381. 32584461 10.1634/theoncologist.2019-0951 PMC7485367

[29] Muchuweti D , Nyandoro G , Muguti E , Muchaziwepi T . Factors contributing to delayed breast cancer presentation: a prospective study at parirenyatwa group of hospitals, Harare, Zimbabwe 2010–2013. JCTI. 2017;5:1‐10.

[30] Cubasch H , Dickens C , Joffe M , et al. Breast cancer survival in Soweto, Johannesburg, South Africa: a receptor‐defined cohort of women diagnosed from 2009 to 11. Cancer Epidemiol. 2018;52:120‐127. 29306221 10.1016/j.canep.2017.12.007 PMC6127863

[31] Gakwaya A , Kigula‐Mugambe JB , Kavuma A , et al. Cancer of the breast: 5‐year survival in a tertiary hospital in Uganda. Br J Cancer. 2008;99:63‐67. 18577991 10.1038/sj.bjc.6604435 PMC2453032

[32] Mushonga M . Biomarkers in Breast Cancer: quantifying discordance with best practice when hormone receptor status is an extravagance. SA J Oncol. 2020;4:a134.

[33] Foroudi F , Tyldesley S , Walker H , Mackillop WJ . An evidence‐based estimate of appropriate radiotherapy utilization rate for breast cancer. Int J Radiat Oncol Biol Phys. 2002;53:1240‐1253. 12128126 10.1016/s0360-3016(02)02821-3

[34] Danielson B , Winget M , Gao Z , Murray B , Pearcey R . Palliative radiotherapy for women with breast cancer. Clin Oncol. 2008;20:506‐512. 10.1016/j.clon.2008.04.013 18524556

[35] Makanjuola SBL , Popoola AO , Oludara MA . Radiation therapy: a major factor in the five‐year survival analysis of women with breast cancer in Lagos, Nigeria. Radiother Oncol. 2014;111:321‐326. 24746579 10.1016/j.radonc.2014.03.013

[36] Sung H , DeSantis CE , Fedewa SA , Kantelhardt EJ , Jemal A . Breast cancer subtypes among Eastern‐African‐born black women and other black women in the United States. Cancer. 2019;125:3401‐3411. 31190337 10.1002/cncr.32293

[37] Jiagge E , Chitale D , Newman LA . Triple‐negative breast cancer, stem cells, and African Ancestry. Am J Pathol. 2018;188:271‐279. 29137951 10.1016/j.ajpath.2017.06.020

[38] Youngblood VM , Nyirenda R , Nyasosela R , et al. Outcomes and prognostic factors for women with breast cancer in Malawi. Cancer Causes Control. 2020;31:393‐402. 32124187 10.1007/s10552-020-01282-4 PMC7115156

[39] Eng A , McCormack V , dos‐Santos‐Silva I . Receptor‐defined subtypes of breast cancer in indigenous populations in Africa: a systematic review and meta‐analysis. PLoS Med. 2014;11:e1001720. 25202974 10.1371/journal.pmed.1001720 PMC4159229

[40] Poudyal BS , Giri S , Tuladhar S , Neupane S , Gyawali B . A survey in Nepalese patients with acute leukaemia: a starting point for defining financial toxicity of cancer care in low‐income and middle‐income countries. Lancet Haematol. 2020;7:e638‐e639. 32853583 10.1016/S2352-3026(20)30258-1

[41] Iyer HS , Kohler RE , Ramogola‐Masire D , et al. Explaining disparities in oncology health systems delays and stage at diagnosis between men and women in Botswana: a cohort study. PLoS One. 2019;14:e0218094. 31170274 10.1371/journal.pone.0218094 PMC6553768

[42] Schleimer LE , Vianney Dusengimana J‐M , Butonzi J , et al. Barriers to timely surgery for breast cancer in Rwanda. Surgery. 2019;166:1188‐1195. 31466858 10.1016/j.surg.2019.06.021 PMC6861658

[43] Brown CA , Kohler RE , John O , et al. Multilevel factors affecting time to cancer diagnosis and care quality in Botswana. Oncologist. 2018;23:1453‐1460. 30082488 10.1634/theoncologist.2017-0643 PMC6292540

[44] Kohler RE , Gopal S , Miller AR , et al. A framework for improving early detection of breast cancer in sub‐Saharan Africa: A qualitative study of help‐seeking behaviors among Malawian women. Patient Educ Couns. 2017;100:167‐173. 27528411 10.1016/j.pec.2016.08.012 PMC5301948

[45] Tapela NM , Peluso MJ , Kohler RE , et al. A step toward timely referral and early diagnosis of cancer: implementation and impact on knowledge of a primary care‐based training program in Botswana. Front Oncol. 2018;8:187. 29896450 10.3389/fonc.2018.00187 PMC5986942

[46] Rutayisire R , Mutabazi F , Bayingana A , et al. Integration of chronic oncology services in noncommunicable disease clinic in Rural Rwanda. Ann Glob Health. 2020;86:33. 32257833 10.5334/aogh.2697 PMC7101006

[47] Grover S , Chiyapo SP , Puri P , et al. Multidisciplinary gynecologic oncology clinic in Botswana: a model for multidisciplinary oncology care in low‐ and middle‐income settings. J Glob Oncol. 2017;3:666‐670. 29094103 10.1200/JGO.2016.006353 PMC5646885

[48] Pinder LF , Nzayisenga J‐B , Shibemba A , et al. Demonstration of an algorithm to overcome health system‐related barriers to timely diagnosis of breast diseases in rural Zambia. PLoS One. 2018;13:e0196985. 29746541 10.1371/journal.pone.0196985 PMC5945023

[49] Chaouki W , Mimouni M , Boutayeb S , Hachi H , Errihani H , Benjaafar N . Evaluation of multidisciplinary team meeting; the example of gynecological mammary cancers in a tertiary referral center in Morocco. Bull Cancer. 2017;104:644‐651. 28571842 10.1016/j.bulcan.2017.04.004

[50] Brown E , Bartlett J , Chalulu K , et al. Development of multi‐disciplinary breast cancer care in Southern Malawi. Eur J Cancer Care. 2017;26:e12658. 10.1111/ecc.12658 28111860

[51] Kim K , Choi JS , Choi E , et al. Effects of community‐based health worker interventions to improve chronic disease management and care among vulnerable populations: a systematic review. Am J Public Health. 2016;106:e3‐e28. 10.2105/AJPH.2015.302987 PMC4785041 26890177

