# Supplementary figures and images for: Breast cancer in Zimbabwe: patterns of care and correlates of adherence in a national referral hospital radiotherapy center cohort from 2014 to 2018
Source: Cancer Med. 2021 May 10;10(11):3489–98. doi: 10.1002/cam4.3764 (PMC8178482; doi:10.1002/cam4.3764)

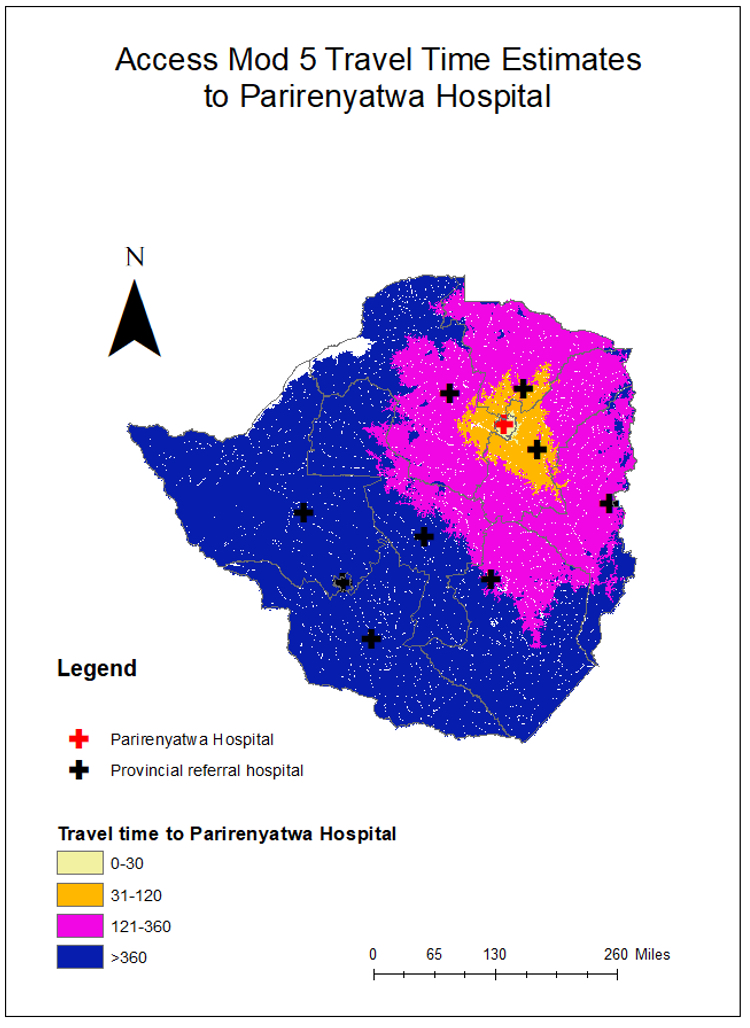

Supplement: Supplementary file 1 — Figure S1 [file CAM4-10-3489-s001.jpg]
